# Rationale, design, and methods of a non-interventional study to establish safety, effectiveness, quality of life, cognition, health-related and work capacity data on Alemtuzumab in multiple sclerosis patients in Germany (TREAT-MS)

**DOI:** 10.1186/s12883-016-0629-9

**Published:** 2016-07-19

**Authors:** Tjalf Ziemssen, Ulrich Engelmann, Sigbert Jahn, Alexandra Leptich, Raimar Kern, Lina Hassoun, Katja Thomas

**Affiliations:** Center of Clinical Neuroscience, Carl Gustav Carus University Hospital, Dresden, Germany; Medical Affairs, Genzyme GmbH, Neu-Isenburg, Germany; Clinical Study Unit, Sanofi-Aventis Deutschland GmbH, Frankfurt am Main, Germany

**Keywords:** Alemtuzumab, Non-interventional trial, Risk-management plan, MSDS3D, Real worl data, Multiple sclerosis

## Abstract

**Background:**

Alemtuzumab, a humanized monoclonal antibody directed against the cell surface glycoprotein CD52, is licensed in Europe since October 2013 as treatment for adult patients with active relapsing-remitting multiple sclerosis (RRMS). In three randomized, rater-blinded active comparator clinical trials studies, alemtuzumab administered in two annual courses, had superior efficacy as compared to subcutaneous interferon beta-1a, and durable efficacy over 5 years in an extension study with a manageable safety profile in RRMS patients. Data on the utilization and the outcomes of alemtuzumab under clinical practice conditions are limited.

**Methods:**

Here we describe the rationale, design and methods of the TREAT-MS study (non-interventional long-Term study foR obsErvAtion of Treatment with alemtuzumab in active relapsing-remitting MS).

**Discussion:**

TREAT-MS is a prospective, multicenter, non-interventional, long-term study to collect data on safety, effectiveness, quality of life, cognition and other aspects from 3200 RRMS patients treated with alemtuzumab under the conditions of real-world clinical practice in Germany.

**Trial registration:**

As non-interventional trial in Germany.

## Background

Multiple sclerosis (MS) is generally considered a primarily T-cell mediated autoimmune disease of the central nervous system (CNS). To date, no cures exist for MS – the disease will progress to a worse stadium with higher disability sooner or later. Only disease course-modifying therapies (DMTs) are available for patients [1]. Treatments for the mild and moderate relapsing remitting MS (RRMS) courses are interferons-β and glatiramer acetate, DMTs which have been used since 20 years [2, 3]. On average, these injectable drugs cut the annual relapses by a third, and they are effective with side effects like flu like symptoms or injection site reactions [3, 4]. In 2013, teriflunomide [5] and in 2014, dimethylfumarate [6] have been introduced as oral agents for RRMS treatment.

Given that first-line therapies might fail to adequately control disease activity in some patients, it has been recommended to switch these patients early to a therapy of higher efficacy more rigorously [7, 8]. Among treatments for (highly) active RRMS offering higher effectivity but also accompanied by significant side effects are DMTs such as fingolimod and natalizumab [9]. Fingolimod reduces the amount of lymphocytes that exit the lymph node by binding to sphingosine-1-phosphate receptors on the cell surface. While annual relapse rates (ARRs) are reduced by more than half [10], cardiac side effects and macular edemas are among the side effects [11]. Natalizumab, a humanized monoclonal anti-α4-integrin antibody, prevents lymphocytes’ crossing the blood-brain barrier [12]. It was shown that it can reduce ARRs by 68 %; however, an especially dangerous adverse effect of natalizumab is progressive multifocal leukoencephalopathy (PML), a brain infection by the John Cunningham (JC) virus [13]. For this complication, a lethality of 20 % in MS patients treated with natalizumab has been reported [14].

### Alemtuzumab

Alemtuzumab (Lemtrada®, marketed by Genzyme) has been approved in Europe 2013 and is marketed as a treatment for RRMS with active disease defined by clinical or imaging features [15]. In the USA, the drug has been approved in November 2014 for RRMS and PRMS treatment, but only for patients who did not have a satisfying response to two or more drugs (i.e. for second-line therapy).

Alemtuzumab is a humanized monoclonoal antibody against the lymphocyte surface protein CD52 [16]. CD52 covers about 5 % of the entire surface of lymphocytes; apart from them, it occurs on cells as diverse as macrophages and endothelial cells [17]. After binding of alemtuzumab to CD52, lymphocytes are destroyed either by complement-induced or antibody-dependent cell-mediated cytotoxicity [16, 18, 19]. As a consequence it is assumed that B- and T-cell repopulation takes place [20] by which – compared with the pre-treatment stage – the proportions of lymphocyte subgroups are shifted; the numbers of regulatory T cells and memory B- and T-cells are increased, and cell populations of innate immunity are also affected [21].

Overall, alemtuzumab appears to re-organize the immune repertoire, which manifests in the special kinetics of immune cell population, the increased production of antiinflammatory cytokines, and last but not least the very long duration of action [18, 22].

Three randomized, rater-blinded clinical trials about the effectiveness of alemtuzumab in MS treatment, using an effective comparator drug, have been performed, CAMMS223 [23], CARE-MS I [24], and CARE-MS II [25]. Administering 12 mg alemtuzumab per day, CAMMS223 and CARE-MS I showed a 69 and 55 % higher reduction of relapses than interferon-β 1a (IFNB-1a). Long-term effectivity of alemtuzumab was also superior compared with IFNB-1a: CARE-MS II showed a reduction of the sustained accumulation of disability (SAD) within 6 months of 42 % and a reduction of relapses per year of 49 %. SAD reduction in a 5-year perspective was 69 %, and reduction of relapses 66 % in this long-term outlook [26]. In sum, alemtuzumab drastically slowed down progression of MS.

Important adverse effects elicited by alemtuzumab are secondary autoimmune reactions, in particular (for unknown reasons) reactions targeting the thyroid gland [27, 28]. In the studies, 26, 18 and 16 %, respectively, of patients were diagnosed with thyroid autoimmune disease (AID) [15]. Furthermore, a few cases of Goodpasture Syndrome were observed [29]. In this AID, the basement membrane of kidneys is attacked, which can lead to kidney failure. Idiopathic thrombocytopenic purpura has also been described as a serious adverse effect, causing severe hemorrhages [30–32]. Last, like with natalizumab, reactivation of the JC virus can lead to potentially lethal PML which has been reported in hematological diseases where alemtuzumab was often administered concurrently with immunosuppressive therapy [33, 34]. Up to now, there was only one carry over PML MS case switching from natalizumab to alemtuzumab.

Regular tests before, during and after administration of alemtuzumab have been recommended to recognize and terminate further development of such serious adverse drug reactions [28, 35]. The implementation of a systematic safety monitoring program allows for the early detection and management of autoimmune and other known events [28].

### Rationale of the TREAT-MS study

Randomized controlled trials (RCTs) are the “gold standard” for generating evidence of the efficacy and safety of a drug. However, enrolment criteria, timelines, and atypical comparators of RCTs limit relevance to standard clinical practice. Real-world data (RWD) provide longitudinal information on comparative effectiveness and tolerability of drugs, as well as their impact on resource use, medical costs, pharmacoeconomic outcomes, and patient-reported outcomes [36, 37]. Regarding alemtuzumab, the collected data from clinical studies provide a sound body of evidence on the efficacy and the safety profile in the treatment of RRMS. However, data on the utilization and the treatment outcomes of the drug under clinical practice conditions are limited to few reports on small cohorts [38, 39].

Here we describe the rationale, design and methods of the recently established non-interventional open, uncontrolled, prospective, multicenter, and long-term study TREAT-MS (non-interventional long-**T**erm study fo**R** obs**E**rv**A**tion of **T**reatment with Alemtuzumab in active relapsing-remitting MS).

The main goal of TREAT-MS is to establish a broader real-world dataset on the utilization and safety, effectiveness, quality of life and other aspects of the drug in everyday clinical practice. The study encompasses a risk management plan to recognize and counter each occurring adverse effect as early as possible, which will also support physicians who treat MS patients with alemtuzumab in their daily clinical practice [28]. TREAT will additionally investigate how the risk management plan is transferred into clinical practice which is crucial for alemtuzumab with necessary longterm monitoring [40].

## Methods/Design

### Study design

TREAT-MS is a prospective, multicenter, non-interventional long-term study. Data are collected from around 300 neurologists in specialized MS centers (clinical centers or outpatient departments) in all parts of Germany. Patients are eligible for documentation, if they fulfill the EMA label. The study does not stipulate any diagnostic or treatment procedures.

### Study population

Alemtuzumab has not been administered to minors (<18 years). Patients have received a diagnosis of active RRMS from their neurologists. As non-interventional study (NIS) no specific inclusion resp. exclusion criteria apply in order to avoid selection bias in this real world study beyond the relative or absolute contraindications of the alemtuzumab EMA label [41].

### Study flow

**Fig. 1:**
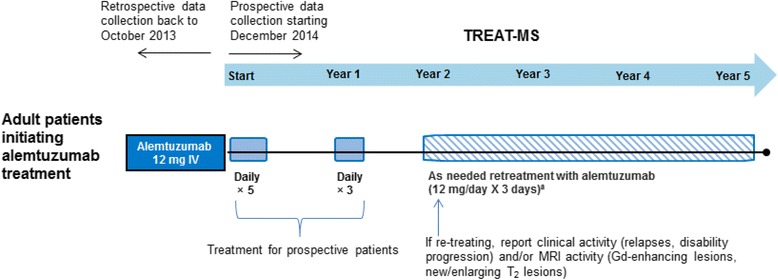
Study design

Figure 1 and the Table 1 provide an overview on the study flow and the items to be documented. At the baseline visit, current and retrospective data on the patient and MS are collected; thereafter patients are followed up in a prospective way. All patients will be monitored for 60 months after the first administration altogether, and for almost 48 months after the second administration of alemtuzumab to vouchsafe a complete realization of the risk management plan. Demographic and clinical data of participants are gathered from medical examinations and other sources (e.g. MS nurse). Information about patient-reported outcomes is completed by participants during their visits in clinics and centers in the presence of a health care professional. Neurologists and MS nurses will be guided by the MSDS 3D-Lemtrada-TREAT-MS module through the entire management of treatment, including monitoring of the first and second infusions, necessary examinations, and regular laboratory screenings.

**Table 1:** Study schedule

			Control examination: month after start of treatment											
			1	2	3	4	5	6	7	8	9	10	11	12
			13	14	15	16	17	18	19	20	21	22	23	24
		Treatment	25	26	27	28	29	30	31	32	33	34	353	36
		1st infusion	37	38	39	40	41	42	43	44	45	46	47	48
	baseline	2nd infusion	49	50	51	52	53	54	55	56	57	58	59	60
Singed declaration of informed consent	x													
Patient characteristics (age and sex)	x													
MS anamnesis														
- Diagnosis	x													
- Date of first diagnosis (RRMS)	x													
- Number of relapses in the last year and the year before	x													
- Comorbidities including fatigue and depression	x													
- Previous MS basis therapy	x													
Relapses during observation time														
- Number	x		x	x	x	x	x	x	x	x	x	x	x	x
- Number of relapses treated with cortisone	x		x	x	x	x	x	x	x	x	x	x	x	x
EDSS (Expanded Disability Status Scale)	x				x			x			x			x
SDMT (Symbol Digit Modality Test)	x							x						x
PRIM US (Patient-Reported Indices for MS)	x							x						x
EuroQol (ED-SD)	x							x						x
Responding to treatment/clinical evaluation														
CGI (Clinical Global Impressions Scale):														
- Details provided by the physician	x							x						x
- Details provided by the patient	x							x						x
Economic parameters														
WPAI (Work productivity and Activity Impairment Questionnaire)	x							x						x
Examinations before and after therapy with Lemtrada^®^														
Pre-existing illnesses	x													
Bodily examination	x													
Total and differential blood count	x	x	x	x	x	x	x	x	x	x	x	x	x	x
Serum creatine level		x	x	x	x	x	x	x	x	x	x	x	x	x
Urine status		x	x	x	x	x	x	x	x	x	x	x	x	x
Thyroid gland function (TSH)	x	x			x			x			x			x
Vaccination status	x													
Infections	x													
HIV infection	x													
Test for tuberculosis	x													
Test for hepatits B and C	x													
Tests for varicella-zoster virus	x													
Test for JC virus	x													
Contraception/exclusion of an existing pregnancy	x		x											
Adverse events since therapy start			x	x	x	x	x	x	x	x	x	x	x	x
MRI (new T2 or Gd(+)-lesions)	x		if MRT examinations are performed routinely											

Baseline at least 6 weeks prior to first alemtuzumab injection, if vaccinations need to be made

#### Documentation by clinicians

##### Treatment preparations

Taking into consideration the well-known side-effects and contraindications of alemtuzumab, a detailed patient history and physical examination should be performed to exclude possible contraindications [28]. Recommended lab tests before starting treatment with alemtuzumab should be done before every alemtuzumab infusion later on. The vaccination status is checked at baseline. Lacking vaccinations (e.g., varicella) are to be administered no later than six weeks before start of alemtuzumab treatment. Tests for various infectious agents (tuberculosis, hepatitis B and C, varicella-zoster and JC virus) are performed to avoid infectious disease complications after infusion.

A specific interest of TREAT study will be the different pretreatment status of the patients as the strategy how to initiate the treatment with alemtuzumab depends on pretreatment status of the patient. As direct switching from interferon-beta or glatiramer Acetate to alemtuzumab is possible, prior therapy with natalizumab represents a special situation as the switching protocol depends on the actual PML risk which is closely linked to the JCV status and natalizumab treatment duration [12, 28].

##### Disease progression and clinical monitoring

Before commencement of treatment, history and disease course of the patient’s MS, including previous therapeutic interventions, are assessed. In particular, the number of relapses in the last year and the year before and comorbidities are documented. The number of relapses is an effectiveness criterion in this study, and thus closely followed up. A relapse is defined as an episode of neurological symptoms that happens at least 30 days after any previous episode began, lasts at least 24 h and is not attributable to another cause and occurs in the absence of an infection or fever. It has to be accompanied by either new clinical signs, i.e. changes in the neurological examination, or an increase in the disability (EDSS) score. If magnetic resonance tomography (MRI) examinations are performed routinely, at every visit results are documented. This includes number of lesions in T2-weighted MRI and gadolinium-enhancing lesions due to analysis of the local radiologist.

##### Alemtuzumab application

Alemtuzumab infusion is documented in a detailed way including concomitant medication and adverse events.

##### Laboratory monitoring

Total and differential blood count, serum creatinine and urine status are evaluated every month after alemtuzumab infusion to gain reference values for measuring the impact of alemtuzumab administration on these health parameters. In addition, thyroid function is assessed every 3 months using TSH. All tests are repeated for 4 years after the last alemtuzumab infusion, and the results are documented in the system.

Further instruments applied by physicians include the Expanded Disability Status Scale (EDSS) and the Clinical Global Impression (CGI) (severity) until termination of the observation phase.

##### Expanded disability status scale

The EDSS is a clinician-rated scale based on neurological history and physical examination, which is used to determine the degree of neurological disability in patients with multiple sclerosis [42–44]. As part of the EDSS Neurostatus, eight functional systems are assessed: pyramidal, cerebellar, brainstem, sensory, bowel and bladder, visual, cerebral and other. The rating is performed by certified health care professionals, most usually a neurologist. It normally takes 20-30 min to complete the rating. The EDSS gives a score from 0 (normal neurological examination) to 10 (death from multiple sclerosis), with half points from 1 upwards. In the TREAT study, EDSS and functional system scores are documented.

##### Clinical global impression

The CGI, developed by the National Institute of Health originally for use in psychiatry, is a three-item scale used to assess treatment response [45]. The present study uses item 2 only, on seven-point scale (1 = very much improved to 7 = very much worse). The CGI is robust, simple (clinically understandable), and sensitive to change.

#### Patient-related self-reported outcomes

The study puts great emphasis on the documentation of patient-related outcomes including functionality, quality of life and ability to work. Thus, patients are requested to fill out various questionnaires at inclusion and at 6-month intervals thereafter.

##### Symbol digit modality test

The SDMT, developed by Wechsler et al., is a neuropsychological test measuring attention [45]. It is brief, easy to administer, and has demonstrated remarkable sensitivity in detecting not only the presence of brain damage, but also changes in cognitive functioning over time and in response to treatment. The SDMT involves a simple substitution task: Using a reference key, the test taker has 90 s to pair specific numbers with given geometric figures. Responses can be written or given orally, and administration time is just five minutes for either response mode.

##### Patient-reported outcome indices for MS

PRIMUS, published in 2009 by Galen Research [46], is a disease specific patient-reported outcome questionnaire that measures quality of life (QoL) of MS patients. The questionnaire consists of three scales: quality of life (22 questions), symptoms (8 questions) and activity status (15 questions), which can either be used by the patient together or as standalone measures. On the QoL scale, questions are to be answered with yes or no, and items are summed to yield a total score ranging from 0 to 22 (with high scores indicating low QoL). The activity limitations scale contains 15-items describing specific physical tasks, and respondents rate the degree to which they are able to perform the tasks on a three point scale. Here, items are summed to give a total score that can range from 0 to 30, with higher scores representing greater activity limitation. Both scales have been shown to be unidimensional and to have good reproducibility and validity in a number of languages [47].

##### Euro-Qol 5D-3 L

The EQ-5D questionnaire, developed by the EuroQol group [48], is a standardized measure of health status, applicable to a wide range of health conditions and treatments. The descriptive system of health-related quality of life states consists of five dimensions (mobility, self-care, usual activities, pain/discomfort, anxiety/depression) each of which can take one of five responses. The responses record five levels of severity (no problems/slight problems/moderate problems/severe problems/extreme problems) within a particular EQ-5D dimension. In addition, self-assessed quality of life is measured using a 0–100 visual analogue scale. The EQ-5D provides a simple descriptive profile and a single index value for health status that can be used in the clinical and economic evaluation of health care as well as in population health surveys [49].

##### Work productivity and activity impairment questionnaire

The WPAI:MS questionnaire consists of 6 questions (1 = currently employed; 2 = hours missed due to health problems; 3 = hours missed other reasons; 4 = hours actually worked; 5 = degree health affected productivity while working (using a 0 to 10 Visual Analogue Scale (VAS)); 6 = degree health affected productivity in regular unpaid activities (VAS). The recall period for the questions 2 to 6 is seven days. Thus, the questionnaire measures absenteeism, presenteeism as well as the impairments in unpaid activity because of health problems during the past seven days [50]. It has been validated to quantify work impairments for numerous diseases such including asthma, psoriasis, irritable bowel syndrome, ankylosing spondylitis and Crohn’s disease. In addition, the WPAI questionnaire has been used to compare work impairments between treatment groups in clinical (studies and) trials or between subjects with different disease severity levels.

##### Adverse events reporting

At every visit, patients are asked for the occurrence of adverse events (AEs). As alemtuzumab is a product under special observation (due to its novelty and immunological effects), each adverse effect occurring during alemtuzumab therapy must be reported, irrespective of a causal connection attributed to alemtuzumab. Time, duration, intensity and outcomes of each AE are documented. Further, the treating physician is asked to assess in terms of a causal connection to alemtuzumab treatment. Severe adverse events are life-threatening or lead to death, hospitalization, to lasting or severe disability, incapacity or congenital anomaly. Adverse events are documented locally in the MSDS 3D-Alemtuzumab-TREAT-Module (see below) and sent automatically to the pharmacovigilance department of Sanofi-Aventis/Genzyme. A list of AEs of special interest is presented in Table 2.

**Table 2:** Adverse events of special interest

Adverse Event	Symptom/finding/disease
Pregnancy	
Symptomatic overdose	
Cancer	
Cervical dysplasia	
Other autoimmune disease	
Infusion-associated reactions	Anaphylactic reactions
Infections	Opportunistic infections, disseminated infections
Diseases of the blood and lymphatic system	Leukopenia, Lymphocytopenia, Thrombozytopenia, **Idiopathic thrombocytopenic purpura (ITP),** Agranulocytosis, Anemia
Liver disease	Elevated transaminases
CNS diseases	**Progressive multifocal leukoencephalopathy (PML)**
Renal diseases	Glomerulonephritis
	**Nephropathies** (e.g. Goodpasture Syndrome)

Particular attention should be given to diseases highlighted **in bold**

##### Risk Management Plan (RMP)

For prescribing doctors and medical specialists who participate in the care for MS patients treated with alemtuzumab, a risk management plan was developed by the drug manufacturer, and approved by the regulatory authorities. It informs precisely about the risks and possible severe adverse effects which can occur during alemtuzumab treatment. Moreover, information is provided to physicians about the required regular tests to ensure clinical vigilance. The documentation system MSDS ^3D^ (see next section) will be used to document all data from the patient according the RMP and expert information for alemtuzumab.

#### MS documentation system for physician, nurse and patient (MSDS^3D^)

Due to the chronicity of MS and its long-term treatment including a post-treatment observation phase, an electronic data recording system which captures all steps of the treatment on a timeline and allows interaction of the participants of the treatment process (physician, nurse, and patient) is very useful or even required for optimal care. Also, large amounts of data arise in the treatment process and should be managed in a systematic way [51].

For these tasks, the MSDS^3D^ software has been developed at Dresden University of Technology (Germany) [52, 53]. It emerged from MSDS Clinic which gathers the personal data of the patient, her/his treatment details and disease course, data from clinical examinations and clinical scores [54]. The software interface presents all procedures in clickable boxes which lead to data entry menus. Then, upon authorization, a MS nurse or neurologist can enter the relevant data (e.g., EDSS, adverse effects).

MSDS^3D^ provides a standardized management and documentation for treatment and disease course of the patient; it can also be used to import data into various database systems as MS BASE [55]. In addition to the features of MSDS Clinic, information can be directly shared with the patient and feedback can be received from him. The patient can deliver information by using a touchscreen in the neurological center, or he can log into the system from his home computer to enter information or reply to queries. For example, by solving a questionnaire about the symptoms of adverse effects in immunomodulatory treatments of MS the patient can help detect severe AEs such as PML or Goodpasture Syndrome early [56].

**Fig. 2:**
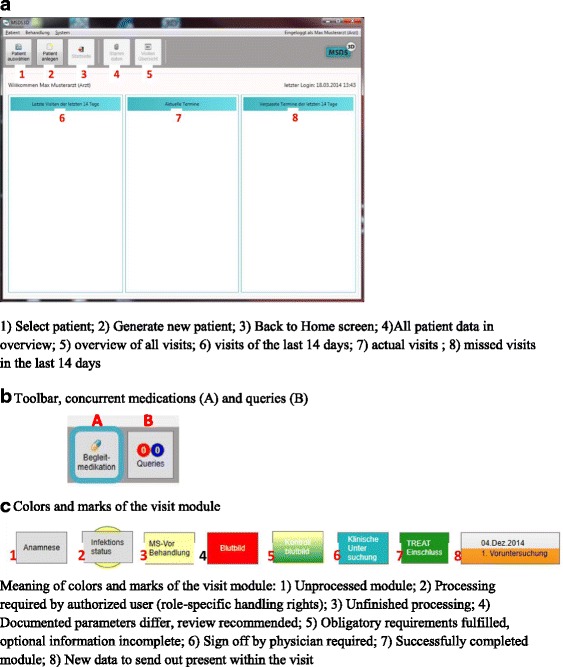
**a** to **c**. MSDS 3D TREAT-MS module: start page

**Fig. 3:**
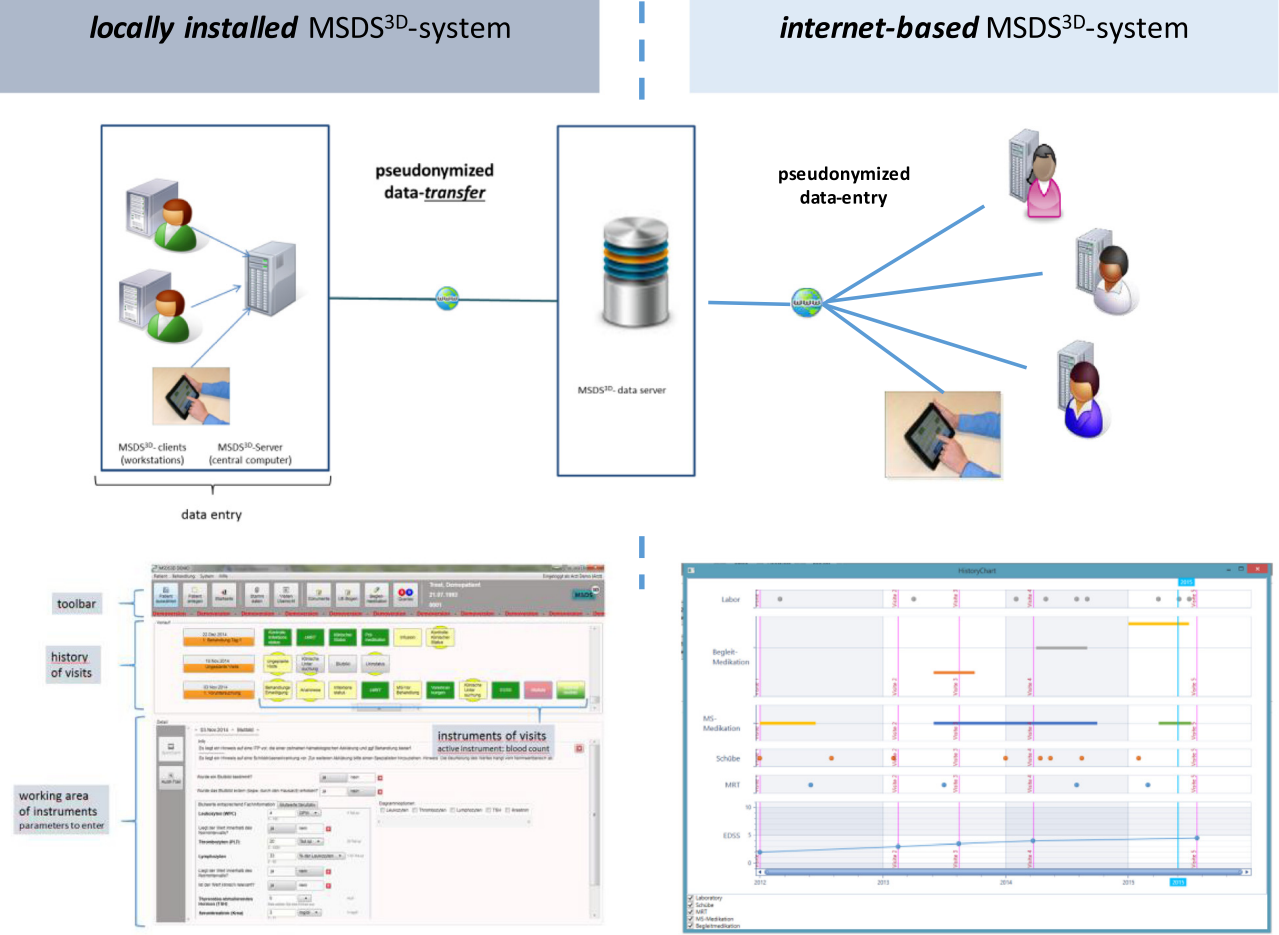
MSDS^3D^ data transfer to the TREAT DATABASE server via internet-based or locally installed MSDS^3D^ system

In addition to MSDS^3D^, the online-based MSDS^3D^-TREAT electronic case report form (eCRF) (Fig. 2a to c) can be used to document patient data, the local MSDS^3D^ software and the MSDS^3D^ web tool are linked to the same database (Fig. 3). These are labelled with a code that does not permit identification of the patient; only physicians or people authorized by the project administration possess access rights to this code.

The MSDS^3D^ interface introduces a feature known from eCRFs. The user sees a vertical timeline, while the procedures which have to be executed during the treatment process are arranged horizontally and shown as clickable ‘procedure boxes’. These lead to input menus for the procedure which is to be executed, e.g. EDSS screening or registration of adverse effects.

Moreover, MSDS^3D^ can create physician’s reports either through entering free text or by using text modules. The system was modified to document immunomodulatory treatments such as alemtuzumab, natalizumab and fingolimod. Before a drug is administered, e.g. as an infusion, the system asks for all relevant health checks, and after all checks have been performed successfully, the treatment is authorized by the neurologist. In the visit module, specific instruments (categorized tasks) are attributed to every visit. Color marks and other signs point to the processing stage of the tasks. In addition, the toolbar contains buttons for concurrent medications and queries about data documented within the system. Red and blue colors show open or answered queries, respectively.

Different MS treatment cohorts have been already followed up using MSDS^3D^ (eg. Fingolimod [57, 58], Natalizumab [56, 59, 60]) which will allow comparisons between different treatment groups. Identification and mitigation of biases and careful consideration of study power are key factors for designing appropriate RWE studies. Various biases exist, which require careful consideration in selecting appropriate comparators, patient populations, data sources, outcomes, and statistical analyses [36].

#### Data management and statistical aspects

Data management has been described in a separate data management plan, which includes a data validation plan. A population size of 3200 patients means that adverse events with an incidence of 1:1068 or more will occur at least once in the alemtuzumab patient population.

For the portion of patients with a premature termination of the risk management plan, 95 % confidence intervals according to the method of Clopper and Pearson are determined. Proportional hazards model will be used to assess whether certain patient subgroups are more likely than other to terminate the RMP early. In general, for continuous variables numbers of patients, mean and standard deviation, the five point summary (minimum, lower quartile, median, upper quartile, maximum) and possibly further appropriate percentiles will be determined. For categorical variables absolute and percentage frequencies will be calculated.

For means and estimated probabilities – and, if significant, for further parameter as well – 95 % confidence intervals will be determined. The evaluations will also be performed for subgroups which are defined before in the statistical analysis plan. Depending on the number of subgroups defined in the plan the confidence level will possibly be raised. The evaluations of the used questionnaires SDMT, EQ-5D, PRIMUS, CGI-S (versions for physician and patient) and WPAI are performed according to validated and published algorithms. All statistical evaluations are of exploratory nature.

##### Timelines

The study started in November 2014 and recruitment is planned until December 2016, however, it will be stopped as soon as a sufficient number of patients have been enrolled. Observation of patients and documentation will continue until September 2022.

## Discussion

In recent years, innovative immunotherapies have offered new treatment options, better disease control and improved the quality of life in patients with MS. With the introduction of natalizumab in 2006 and fingolimod in 2011 as therapies for RRMS, safety aspects became more prominent. In order to address those comprehensively, clearly defined cooperations between MS specialists and doctors from other disciplines, such as radiologists, cardiologists or ophthalmologists needed to be established. In 2013, with teriflunomide and alemtuzumab, further immunotherapeutics for MS were approved, that require before and during application regular check-ups to ensure safe use. The most extensive catalogue of requirements for pharmacovigilance arises in the application of the new anti-CD52  antibody alemtuzumab, which has received a broad labelling in Europe which does not exclude first-line use.

On top of the body of evidence from 1500 patients that received alemtuzumab in the randomized controlled trials (CAMMS223 [23], CARE-MS I [24] and CARE- II [25]), the clinical experience with the agent has substantially increased since market introduction. Nevertheless there are only few reports yet on the long-term use of the drug in the routine of physicians and MS centers. TREAT-MS will fill this gap, and will document physician and patient experience in daily clinical practice for 6 years. Data in this non-interventional study will be collected on a widely unselected patient population eligible for alemtuzumab treatment. It is expected that compared to the clinical studies, patients with more concomitant diseases and/or more concomitant medications will be documented. Together with the high patient numbers and long follow-up period, a substantial number of patient years will be documented and the option for relevant subgroup analyses provided.

As the registry protocol was developed based on the risk management plan for alemtuzumab, by participation in the study, physicians are reminded about the investigations and precautions which are needed for the safe use of this potent immunological drug [28]. Thus, the study serves the additional purpose to optimize the drug utilization according to the conditions specified by the labelling.

The documentation system MSDS^3D^ has been shown to be efficient to guide physicians through the study procedures and to collect the relevant information in clinical practice and for the use in previous non-interventional studies such as PANGAEA [57]. It interactively collects data, but also assists neurologists in the execution of complex processes required for comprehensive management of MS patients.^50^

The planned number of 3200 patients in TREAT-MS are recruited from centers in all parts of the country and different types of centers (office-based, various types and sizes of hospitals), which makes the study representative for the situation in Germany. Thus – apart from triplicating the number of documented individuals treated with alemtuzumab– the evidence of benefits and risks of alemtuzumab for patients as well as for physicians will appear in much higher resolution and depth when the study ends. A two- or three-times longer post-treatment observation phase (compared with the pre-market trials) with monthly clinical checks will also contribute to this effect and lead to more secure information of the incidence of secondary autoimmune diseases and other adverse effects.

TREAT could serve in future as part of a personalized medicine approach in MS, where MS patients are characterized by a detailed clinical profiling and followed up by prospective longterm documentation [61]. This data can be used to analyze treatment response patterns of alemtuzumab and allow personalized treatment approaches. Predictive models could be implemented using this data [62]. So TREAT data will assist in complex treatment decisions in future. It will be interesting to investigate different baseline profiles (de novo patients, patients escalated from first line therapies, patients switching from escalation therapies) and treatment response and safety of alemtuzumab.

Among the limitations of TREAT-MS as an non- interventional, open study is the absence of blinding, neither on the patients’ nor on the physicians’ side. Further, there is no comparator group. The expectation to treat or be treated with a novel anti-MS drug might lead to a higher amount of reported positive effects than observed in a study with blinded subjects or physicians. Moreover, physicians may assign patients to the study based on the severity of their disease, the observation that they did not respond well to conventional drugs, or the presence of complex comorbidities; this might bias study results. Through such possibly biased allocation of participants, assessing the correlation between treatment and outcomes will be difficult.

## Abbreviations

AE, adverse event; AID, autoimmune disease; ARR, annual relapse rate; CGI, clinical global impression; CNS, central nervous system; CRF, Case report form; DMT, disease course-modifying therapy; EDSS, expanded disability status scale; EMA, European medicine agency; IFNB-1a, Interferon beta-1a; JC, John Cunningham; MRI, magnetic resonance imaging; MS, multiple sclerosis; NIS, non-interventional trial; PML, progressive multifocal leukencephalopathy; QoL, quality of life; RCT, randomized controlled trial; RRMS, relapsing-remitting multiple sclerosis; RWD, real world data; SAD, sustained accumulation of disability; SDMT, symbol digit modality test; TREAT-MS study, non-interventional long-Term study foR obsErvAtion of Treatment with alemtuzumab in active relapsing-remitting MS; TSH, Thyroid-stimulating hormone; WPAI, Work Productivity and Activity Impairment
